# Gamma-Ray Protection Properties of Bismuth-Silicate Glasses against Some Diagnostic Nuclear Medicine Radioisotopes: A Comprehensive Study

**DOI:** 10.3390/ma14216668

**Published:** 2021-11-05

**Authors:** Ghada ALMisned, Hesham M. H. Zakaly, Shams A. M. Issa, Antoaneta Ene, Gokhan Kilic, Omemh Bawazeer, Albandari Almatar, Dalal Shamsi, Elaf Rabaa, Zuhal Sideig, Huseyin O. Tekin

**Affiliations:** 1 Department of Physics, College of Science, Princess Nourah Bint Abdulrahman University, Riyadh 11671, Saudi Arabia; gaalmisned@pnu.edu.sa; 2 Institute of Physics and Technology, Ural Federal University, 620002 Ekaterinburg, Russia; 3 Physics Department, Faculty of Science, Al-Azhar University, Assiut 71452, Egypt; shams_issa@yahoo.com; 4 Department of Physics, Faculty of Science, University of Tabuk, Tabuk 71451, Saudi Arabia; 5 INPOLDE Research Center, Department of Chemistry, Physics and Environment, Faculty of Sciences and Environment, Dunarea de Jos University of Galati, 47 Domneasca Street, 800008 Galati, Romania; 6 Department of Physics, Faculty of Science and Letters, Eskisehir Osmangazi University, Eskisehir 26040, Turkey; gkilic@ogu.edu.tr; 7 Medical Physics Department, Faculty of Applied Sciences, Umm-Al Qura University, Makkah 24381, Saudi Arabia; Oabawazeer@uqu.edu.sa; 8 Medical Diagnostic Imaging Department, College of Health Sciences, University of Sharjah, Sharjah 27272, United Arab Emirates; U00030784@sharjah.ac.ae (A.A.); U16106929@sharjah.ac.ae (D.S.); U16107032@sharjah.ac.ae (E.R.); U16100701@sharjah.ac.ae (Z.S.); 9 Medical Radiation Research Center (USMERA), Uskudar University, Istanbul 34672, Turkey

**Keywords:** shielding parameters, bismuth silicate glasses, radioisotope energies, MCNPX code

## Abstract

This study aimed to perform an investigation for the potential implementation of bismuth silicate glasses as novel shield equipment instead of ordinary shields in nuclear medicine facilities. Accordingly, a group of Bi_2_O_3_ reinforced silicate glass system were investigated and compared with ordinary shields in terms of their gamma-ray attenuation properties in diagnostic nuclear medicine radioisotope energies emitted from ^99m^Tc, ^111^In, ^67^Ga, ^123^I, ^131^I, ^81m^Kr, ^201^Tl, ^133^Xe. Mass attenuation coefficient $(\mu_{m})\;$results for glass samples were calculated comparatively with the XCOM program and MCNPX code. The gamma-ray attenuation parameters such as half value layer (HVL), tenth value layer (TVL), mean free path (MFP), effective atomic number (Z_eff_) were obtained in the diagnostic gamma ray energy range from 75 to 336 keV. To confirm the attenuation performance of superior sample, obtained results were extensively compared with ordinary shielding materials. According to the results obtained, BISI6 glass sample with the highest Bi_2_O_3_ additive has an excellent gamma-ray protection.

## 1. Introduction

Among the healthcare-based studies, medical radiation is still a hot topic for researchers and practitioners in different sub-fields such as nuclear medicine, radiation therapy, and diagnostic radiology. The term of medical radiation has a wide variety of utilization worldwide. Besides the benefits from medical radiation, concerns on exposure to a radiation dose of radiation workers cannot be ignored. Therefore, radiation protection is an essential issue for occupational and public health [1,2,3]. In addition to the personal protective equipment of radiation workers, the protection of radiation sources is also a significant task to be considered. For example, although the majority of diagnostic radiology equipment use ionizing X-rays to acquire anatomical information from patients, radiation doses from CT and X-ray facilities are expected to have a greater effect on the environment and worker during the examination. On the other hand, the situation of nuclear medicine facilities is different in terms of implemented radiation type and structure of the clinical and laboratory environment (i.e., HOT lab). Local and international regulations have determined the utilization of radiopharmaceuticals (RPs) to ensure the safety of the personnel working with the RPs; internal regulations of clinical implementation should be considered. Unlike the radiation technologists of diagnostic radiology, it is worth mentioning that there is another stakeholder such as isotope technician and isotope technologist in nuclear medicine facilities. This is often beneficial for isotope experts to use a ring dosimeter on one or both hands in addition to standard safety equipment if they have high rates of activity to control. This allows workers to monitor radiation doses while working with highly radioactive materials in a safe manner. Lead (Pb) and lead-based materials, on the other hand, are the most often used materials in radiation safety. These include heated laboratory shields, syringe and bottle covers, screened wastes, and covered workstations. Meanwhile, tungsten (W) is the preferred choice for medical and industrial settings that require radiation shielding since it uses less material than lead to provide the same level of absorption. More recent findings have found that lead has harmful effects on both the human population and the environment. The concrete is used to resist electromagnetic ionizing radiation (normal or heavy). Nevertheless, it is only used in buildings, which is very heavy and costly but is no more efficient than usual. Concrete is vulnerable to cracking as it is used, making it translucent and immovable [4,5]. Alloys, minerals, marbles, slag, steel, and polymers have been investigated for their ability to shield against ionizing radiation [6,7,8,9,10,11,12,13,14,15,16]. Among the latest generation shielding materials, glass materials have become a strong alternative to the negativity of Lead and concrete materials used in protection against radiation. Cheapness, lightweight, easy-to-form manufacturing compared to Lead and concrete materials, and most of all do not affect living conditions adversely. There are number of investigations on gamma-ray shielding properties of glass materials. Structural and optical studies belonging to TeO_2_ and B_2_O_3_ glasses containing bismuth are commonly found in the literature [17,18]. However, this study was based on SiO_2_ glasses containing Bi_2_O_3_ [19,20]. El Batal studied various properties of bismuth silicate glasses containing Bi_2_O_3_ at a ratio higher than 55% and also reported the change in spectroscopic properties after gamma irradiation [19]. The change in conduction mechanism of bismuth silicate glasses containing high ratios of bismuth with doping with titanium was the subject of another study [20]. Bi_2_O_3_ glass structures exhibit increased handling properties as ionomers for radioactivity resistance but are especially well suited for electronic application and ceramic materials [21,22,23,24] compared to Pb-based glasses [25,26]. On the other hand, there has been an increased glass network communication in the tellurite glasses in the presence of heavy metal oxide in the tellurite glasses [27]. In this study, which was based on literature studies, 6 bismuth silicate glass samples were envisioned according to their Bi_2_O_3_ content within the range of 20–70% mole and were tested for their attenuation against diagnostic energy in nuclear medicine for the purposes of shielding effectiveness. The investigated radioisotopes and their gamma-ray energies can be listed as follow.
• Tc-99m140 keV• In-111172,247 keV• Ga-6793,185,300 keV• I-123159 keV• I-131364 keV• Kr-81m190 keV• Tl-20175,167 keV• Xe-133364 keV

In addition, obtained results have been compared with traditional shielding materials as well as with available shielding materials in the literature. The study’s key goal was to search for nuclear medicine-specific shielding products that can replace lead and concrete-based materials. Accordingly, we hypothesized to report several effects of Bi_2_O_3_ reinforcement on the attenuation of ionizing nuclear radiation types. As a result, the data from each nuclear shielding parameter will be addressed in the analyzed glass samples concerning the increase in Bi_2_O_3_ additive. The results of this large-focused study would have great significance for research on a new generation of radiation-shielding glass shields and their advanced development.

## 2. Materials and Methods

### 2.1. Theoretical Density Calculations


(1) $$\rho =\left(x_{{\mathrm{Bi}}_{2}O_{3}}.\rho_{{\mathrm{Bi}}_{2}O_{3}}\right)+\left(x_{{\mathrm{SiO}}_{2}}.\rho_{{\mathrm{SiO}}_{2}}\right)\;$$

Theoretical densities belonging to Bi_2_O_3_-SiO_2_ glass compositions are given in Equation (1) [28]. In this equation, ρ is the theoretical density of the glass samples, x_i_ is the molar fraction, ρ_i_ is the density value of the chemical substance.

### 2.2. Method of Calculating Radiation Absorption Parameters

Primary gamma-ray intensity drops exponentially because of the Beer–Lambert law: Positioning the attenuator shield between the detector and the source lowers gamma-ray intensity [29,30,31].

(2) $$I=I_{o}e^{-\mu x}$$

In Equation (1), I_o_ depicts the intensity of primary gamma-rays; on the other hand, I represent the intensity of transmitted gamma through the attenuator sample. Moreover, μ indicates the linear attenuation coefficient of the energy of interest. The term x is the thickness of the attenuator sample. Mass attenuation coefficients for glasses can be found using next equation [32,33]:

(3) $$\mathrm{MAC}=\underset{i}{\sum }w_{i}{\left(\mathrm{MAC}\right)}_{i}$$

w_i_: Weight fraction of the i^th^ constitute elements.

A highly accurate calculation of the total atomic cross section (σ_a_) and electronic (σ_t_) and the effective atomic density (σ_a_ dependent on the total molecular cross section (σ_t_) are calculated based on these values.

(4) $$\sigma_{t}=\frac{1}{N_{A}}\underset{i}{\sum }n_{i}A_{i}{\left(\mathrm{MAC}\right)}_{i}$$

(5) $$\sigma_{a}=\frac{1}{N_{A}}\underset{i}{\sum }f_{i}A_{i}{\left(\mathrm{MAC}\right)}_{i}$$

(6) $$\sigma_{e}=\frac{1}{N_{A}}\underset{i}{\sum }\frac{f_{i}A_{i}}{Z_{i}}\;{\left(\mathrm{MAC}\right)}_{i}\;$$

(7) $$Z_{\mathrm{eff}}=\frac{\sigma_{a}}{\sigma_{e}}$$

(8) $$N_{\mathrm{eff}}=\frac{\left(\mathrm{MAC}\right)}{\sigma_{e}}\;$$

n_i_: Number of atoms, A_i_: atomic weight of i^th^ element; Z_i_: atomic number of i^th^ element; f_i_: fractional abundance of i^th^ element; N_A_: Avogadro number

Some attenuators are able to decrease the absorbed radiation level to 1/2: this is called the HVL, and the following equation can be used:

(9) $$\mathrm{HVL}=\frac{\ln\left(2\right)}{\mathrm{LAC}}$$

An absorption of 0.368 of the incident gamma radiation was observed by samples that have a thickness of one mean free path (MFP):

(10) $$\mathrm{MFP}=\frac{1}{\mathrm{LAC}}$$

### 2.3. Monte Carlo Simulations

**Figure 1:**
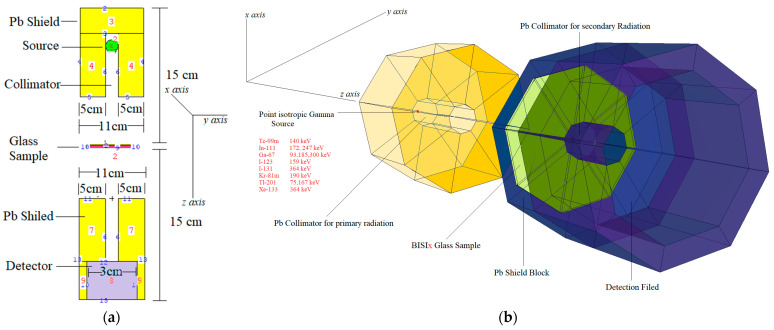
(**a**) Modeled point isotropic radioactive source obtained from MCNPX visual editor; (**b**) 2D view of simulation setup obtained from MCNPX visual editor.

Formal modeling techniques are commonly employed in nuclear shielding tests, commonly employed in numerical assessment. Many simulations can be carried out using Monte Carlo techniques on radiation shielding. Work has been done in MCNPX [34] general-purpose Monte Carlo code to simulate a point isotropic radioactive source has been conducted. Aspherical geometry has been defined as a source. In addition, the gamma-ray energies of isotope were defined for each diagnostic nuclear medicine radioisotope, i.e., ^99m^Tc, ^111^In, ^67^Ga, ^123^I, ^131^I, ^81m^Kr, ^201^Tl, ^133^Xe. In MCNPX simulation, there is no direct outcome to record MAC values but another sub-calculation via analyzing of output file. Firstly, Monte-Carlo simulation data was constructed by adding the key parts, including cell cards, surface cards, and source information in MCNPX input file. Each glass sample was defined considering elemental mass fractions (wt%), material density (g/cm^3^), and geometric form. It is essential to include material properties for radiation interaction issues, including radiation shielding and nuclear protection—the knowledge needed to describe physical quantities defined for an MCNPX input source. Therefore, material definitions of MCNPX input file were performed using the glass properties (see Table 1). The configuration of the modeled devices for gamma-ray transmission studies can be seen in Figure 1. To calculate the average ingested dose, the precise F4 Tally mesh was used. This is a promising technique for measuring average photon flux in a cell [33]. Our models omitted photon and electron energy cutoffs. To begin, the MCNPX code was executed for 10^8^ histories or NPS (number of particle history). In all simulations, the uncertainty associated with MC estimates was less than 1%. The source definition was completed in the INPUT file’s sdef (source definition) section. As a result, the variables si (source probability) and sp (source bias) were defined in terms of the gamma-ray beam distribution from the source. The source was a point source biased toward the glass sample in the direction of experimental gamma ray transmission investigations. The evaluation of the recent MCNPX simulation has been performed by utilizing the D00205ALLCP03 MCNPXDATA package is included of DLC-200/ MCNPDATA cross-section libraries.

## 3. Results and Discussion

In this study, gamma ray attenuation properties of different bismuth silicate glasses were investigated. Samples codes, chemical composition, elemental compositions, and density (ρ) of glass samples can be obtained from Table 1. Sample densities obtained with theoretical method are in compatibility with similar bismuth silicate glass structures found in the literature, yielding experimentally obtained densities [19]. In addition, Inaba reported that theoretically obtained density values were reasonably consistent with experimental density values [35]. According to Table 1, BISI6 has the highest density, and BISI1 has the lowest density. Mass attenuation coefficient $(\mu_{m})\;$results for glass samples encoded BISI1, BISI2, BISI3, BISI4, BISI5, and BISI6 were calculated with the MCNPX [34] code and XCOM [36] program, and the results were obtained in some of the well-known diagnostic nuclear medicine gamma-ray energy range of 75 and 336 keV (see Table 2).

**Figure 2:**
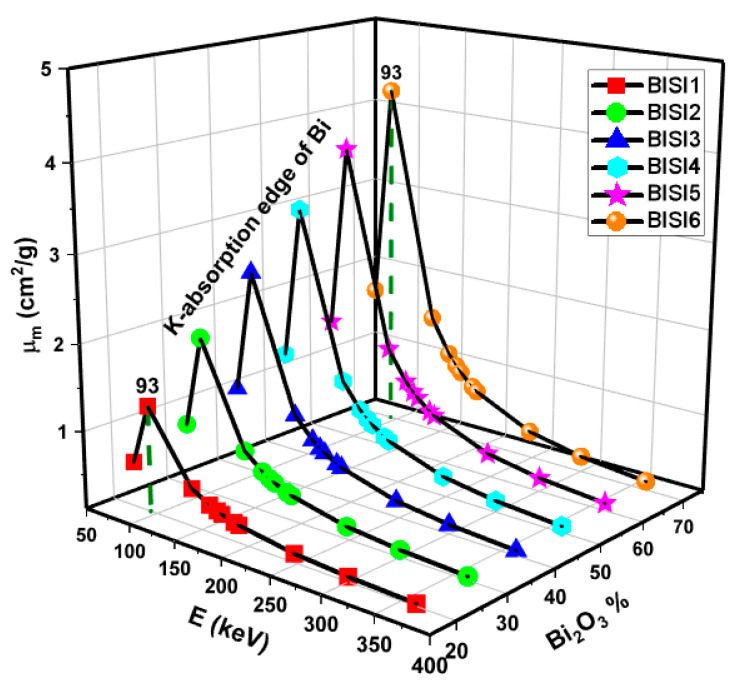
Mass attenuation coefficient (μ_m_) values as a function of photon energy and Bi_2_O_3_ content of glass samples.

The obtained variation trend of mass attenuation coefficients is shown in Figure 2. A sharp peak is observed at 93 keV due to the K absorption edge in the Bi portion for the glasses (as seen in Figure 2). Moreover,${\;\mu }_{m}$ values decrease as the energy value rises from 75 to 336 keV. On the other hand, $\mu_{m}$ increased with the increase of Bi_2_O_3_ additive in the glass composition. The prominent processes above are photoelectric (PE), Compton scattering (CS), and pair-production (PP). The energy changes influenced all the glasses’ energy attenuation coefficients. The sharp decline in $\mu_{m}$ values are caused by the photoelectric effect predominant at low energies. This is due to the microscopic cross-section being linked to the Z^4−5^/E^3.5^ relations. On the other hand, Compton scattering is effective in medium energies with a smooth change. The results showed that the BISI6 sample has the highest mass attenuation coefficients among the investigated glasses. To compare mass attenuation coefficients of BISI6 with ordinary and previously studies shields, a comparison was performed between the materials.

**Figure 3:**
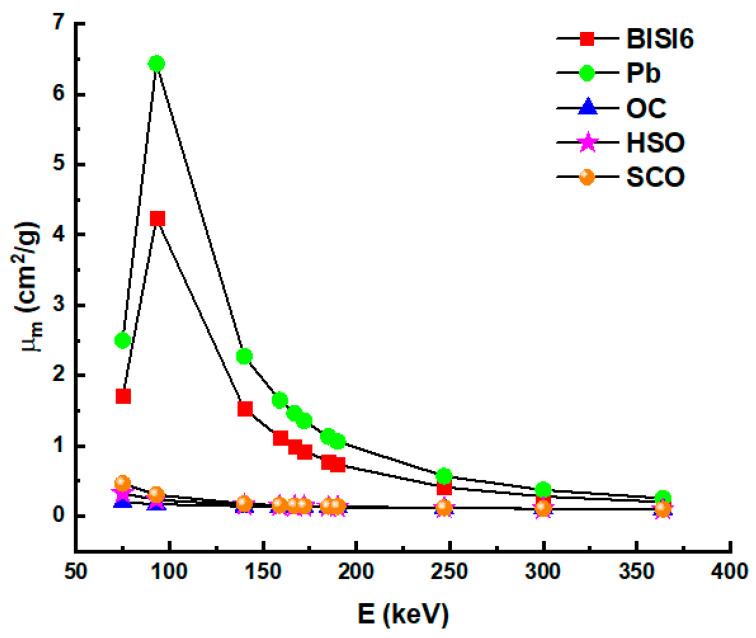
Mass attenuation coefficient (μ_m_) values of BISI6 glass samples Pb, ordinary concrete (OC), hematite-serpentine concrete (HSO), steel-scrap concrete (SCO) as a function of photon energy.

Figure 3 shows the relationship between the mass attenuation coefficient values of BISI6 glass samples with some concrete (OC, HSO, and SCO) samples and Pb as a function of photon energy between 75–336 keV. As shown in Figure 3, the glass sample BISI6 with the highest Bi_2_O_3_ additive is the material closest to the mass attenuation coefficient of lead material. A very critical shielding parameter is also known as HVL. This parameter provides the required thickness to reduce incoming photon energy to its half at a specific energy. Therefore, HVL is very significant, especially during the selection of the most proper shields.

**Figure 4:**
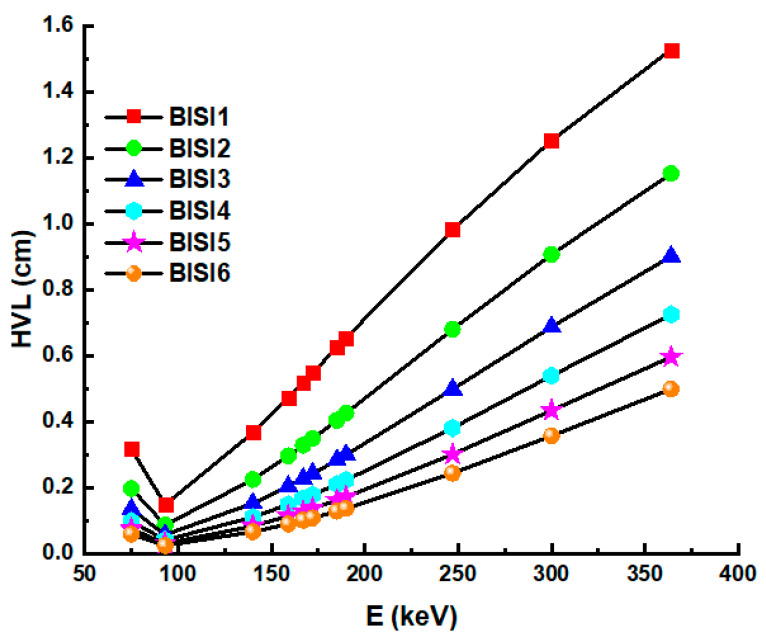
Half value layer (HVL) values as a function of photon energy of glass samples.

Figure 4 shows the energy-dependent variation of the HVL values of all glass materials between the energy region of 75–336 keV. Figure 4 shows that BISI6 has the lowest half value layer values at all gamma-ray energies. Therefore, one can say that the sample with the lowest HVL value has the best protection feature. As seen from the figure, the increase of Bi_2_O_3_ additive and the maximum density decreases the HVL value and increases the gamma attenuation capacity. Therefore, among the glass samples, it is seen from the figures that BISI6 has the best shielding feature. As explained above, this glass sample has the lowest HVL value.

**Figure 5:**
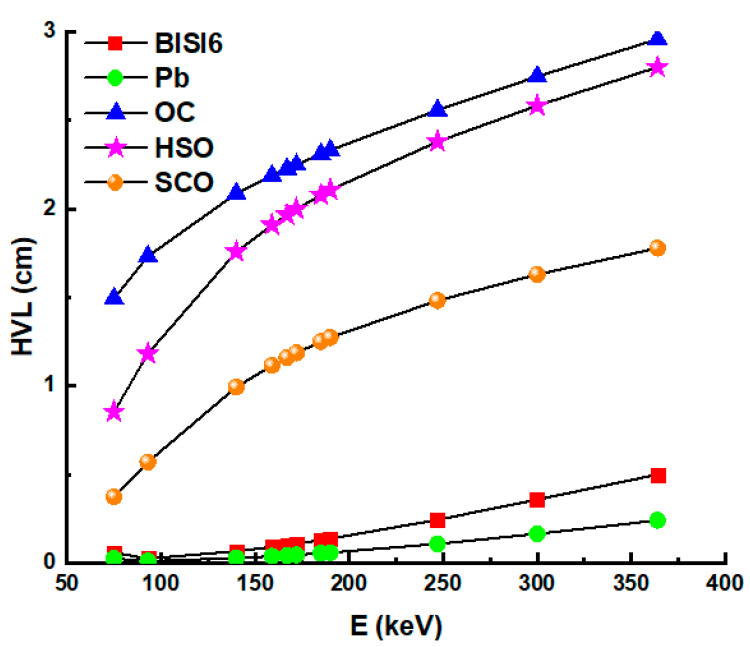
Half value layer (HVL) values of BISI6 glass samples Pb, ordinary concrete (OC), hematite-serpentine concrete (HSC), steel-scrap concrete (SCC) as a function of photon energy.

**Figure 6:**
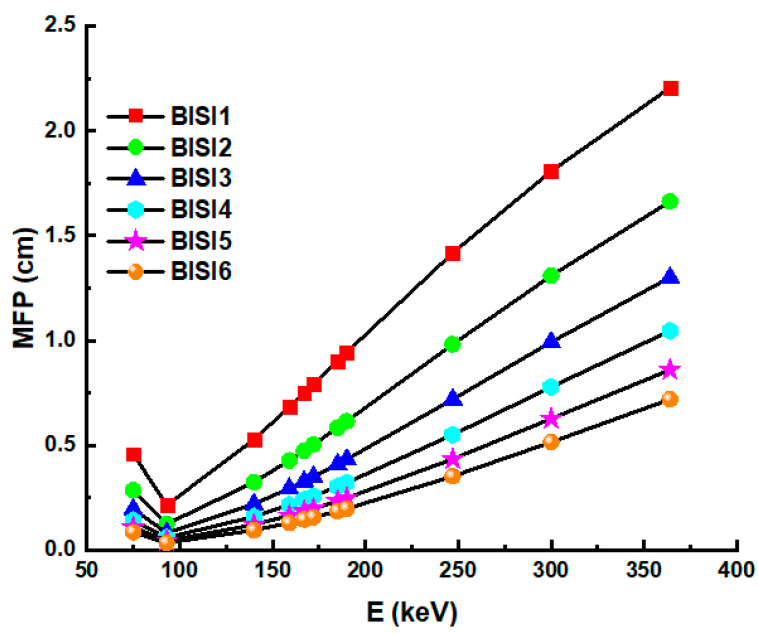
Mean free path (MFP) values as a function of photon energy of glass samples.

In Figure 5, the HVL values of the BISI6 glass sample and the Pb and several concrete (OC, HSC, and SCC) samples were compared. The results showed that BISI6 sample has higher HVL values than Pb but lower than OC, HSC, and SCC [37]. On the other hand, TVL values, which are the calculation of the shielding material thickness required to decrease the intensity of the incoming photons to one-tenth, have also been calculated in the energy range between 75 and 336 keV and shown in Figure 6.

There is an inverse relationship between the mean free path and the linear attenuation coefficient in $\lambda =\frac{1}{\mu }$. Alternately, the linear attenuator with the most significant free path coefficients may be represented as the attenuation value with the lowest attenuation. Thus, strong attenuation characteristics may result in shorter minimum mean free routes. It is plotted in Figure 6 as mean free path difference overall glasses.

**Figure 7:**
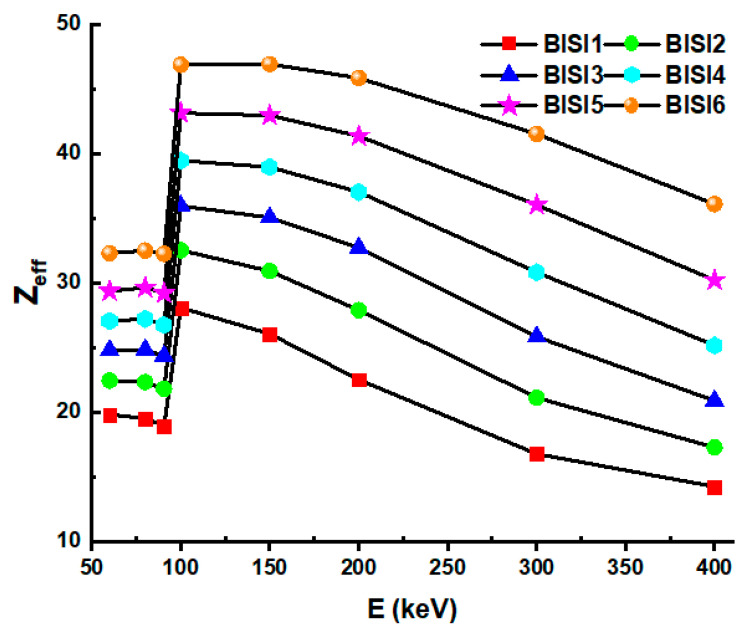
Effective atomic number (Z_eff_) values as a function of the photon energy of glass samples.

We observed that when incident energy rose, the mean free pathways of all the glasses became longer. In addition, however, there were notable variations between the glasses. The findings revealed that BISI6 samples had the shortest mfp values among the investigated samples. Figure 7 shows the Z_eff_ values as a function of incident photon energy. The effective atomic number (Z_eff_) is a measure used to characterize the ionizing radiation reactions of various elemental configurations [11,38,39,40,41]. Energy values are changed from 75 to 336 keV and are shown for all glass samples. Among the glass samples, BISI6 has the highest Z_eff_ value. As it is observed, Z_eff_ values decrease with increasing energy value. The sudden change in Z_eff_ value at low energies (93 keV) is due to the absorption edge K of the Bi element.

## 4. Conclusions

This study aimed to perform a characterization of Bi_2_O_3_ rich silicate glass systems in terms of their availability for utilization as a shield in nuclear medicine facilities. Therefore, gamma-ray energies of used isotopes were defined considering diagnostic nuclear medicine radioisotopes and their gamma-ray energy values. Some types of standard shielding materials, such as Pb and concrete, have significant photon shielding properties. However, some recent investigations and tests found that lead-based products have extreme side effects such as toxicity, poor efficiency, and high price. Therefore, this study attempted to explore eco-friendly alternatives for nuclear medical facilities. Bismuth silicate samples with various bismuth content were tested as new protective material. According to the observations, BISI6 glass has an outstanding radiation shielding property by using the Bi_2_O_3_ additive. Moreover, half-value layers of superior samples encoded BISI6 have been compared with traditional shielding materials such as lead, ordinary concrete (OC), hematite-serpentine concrete (HSC), steel-scrap concrete (SCC) as a function of photon energy. The comparison results showed that the BISI6 sample has significant superiority to ordinary concrete (OC), hematite-serpentine concrete (HSC), steel-scrap concrete (SCC). However, slight differences were obtained between the lead and BISI6 samples. The superiority of lead was slightly more in the range of 75–190 keV gamma-ray energies. On the other hand, the HVL differences between BISI6 and lead slightly increased in the range of 190–364 keV.

## Figures and Tables

**Table 1:** Samples codes, chemical composition, elemental compositions and density (ρ) of glass samples.

Code	Bi_2_O_3_ (mole%)	SiO_2_ (mole%)	O (wt%)	Si (wt%)	Bi (wt%)	ρ (g/cm^3^)
**BISI1**	20	80	0.52529	0.295312	0.179398	3.537
**BISI2**	30	70	0.472505	0.258398	0.269097	4.207
**BISI3**	40	60	0.41972	0.221484	0.358796	4.878
**BISI4**	50	50	0.366935	0.18457	0.448495	5.548
**BISI5**	60	40	0.31415	0.147656	0.538194	6.218
**BISI6**	70	30	0.261365	0.110742	0.627893	6.889

**Table 2:** Comparison of mass attenuation coefficients obtained from MCNPX [34] and XCOM [36].

E (keV)	BISI1		BISI2		BISI3		BISI4		BISI5		BISI6	
	*XCOM*	*MCNPX*	*XCOM*	*MCNPX*	*XCOM*	*MCNPX*	*XCOM*	*MCNPX*	*XCOM*	*MCNPX*	*XCOM*	*MCNPX*
75	0.617	0.619	0.835	0.836	1.053	1.058	1.271	1.273	1.489	1.491	1.707	1.712
93	1.324	1.327	1.906	1.912	2.488	2.491	3.069	3.101	3.651	3.653	4.232	4.238
140	0.535	0.539	0.733	0.735	0.931	0.935	1.130	1.136	1.328	1.334	1.526	1.531
159	0.415	0.417	0.556	0.559	0.697	0.701	0.838	0.841	0.979	0.981	1.121	1.123
167	0.378	0.379	0.501	0.504	0.625	0.627	0.749	0.754	0.873	0.876	0.996	0.103
172	0.358	0.357	0.472	0.475	0.586	0.589	0.700	0.705	0.814	0.815	0.929	0.931
185	0.314	0.317	0.407	0.408	0.501	0.503	0.595	0.601	0.689	0.701	0.783	0.784
190	0.299	0.301	0.387	0.391	0.474	0.471	0.561	0.564	0.648	0.653	0.736	0.738
247	0.199	0.202	0.242	0.245	0.285	0.286	0.328	0.331	0.370	0.372	0.413	0.417
300	0.156	0.157	0.182	0.181	0.207	0.210	0.232	0.235	0.257	0.256	0.282	0.285
364	0.128	0.129	0.143	0.145	0.158	0.159	0.172	0.174	0.187	0.190	0.202	0.205

## Data Availability

Data is contained within the article.
